# Interaction of γ-Fe_2_O_3_ nanoparticles with *Citrus maxima* leaves and the corresponding physiological effects via foliar application

**DOI:** 10.1186/s12951-017-0286-1

**Published:** 2017-07-11

**Authors:** Jing Hu, Huiyuan Guo, Junli Li, Yunqiang Wang, Lian Xiao, Baoshan Xing

**Affiliations:** 1 School of Chemistry, Chemical Engineering and Life Sciences, Wuhan University of Technology, Wuhan, 430070 People’s Republic of China; 2 Institute of Economic Crops, Hubei Academy of Agricultural Sciences, Wuhan, 430064 People’s Republic of China; 3 Stockbridge School of Agriculture, University of Massachusetts, Amherst, MA 01003 USA

**Keywords:** γ-Fe_2_O_3_ nanoparticles, Nano-enabled fertilizer, Foliar spray, Wax, Gene expression

## Abstract

**Background:**

Nutrient-containing nanomaterials have been developed as fertilizers to foster plant growth and agricultural yield through root applications. However, if applied through leaves, how these nanomaterials, e.g. γ-Fe_2_O_3_ nanoparticles (NPs), influence the plant growth and health are largely unknown. This study is aimed to assess the effects of foliar-applied γ-Fe_2_O_3_ NPs and their ionic counterparts on plant physiology of *Citrus maxima* and the associated mechanisms.

**Results:**

No significant changes of chlorophyll content and root activity were observed upon the exposure of 20–100 mg/L γ-Fe_2_O_3_ NPs and Fe^3+^. In *C. maxima* roots, no oxidative stress occurred under all Fe treatments. In the shoots, 20 and 50 mg/L γ-Fe_2_O_3_ NPs did not induce oxidative stress while 100 mg/L γ-Fe_2_O_3_ NPs did. Furthermore, there was a positive correlation between the dosages of γ-Fe_2_O_3_ NPs and Fe^3+^ and iron accumulation in shoots. However, the accumulated iron in shoots was not translocated down to roots. We observed down-regulation of ferric-chelate reductase (FRO2) gene expression exposed to γ-Fe_2_O_3_ NPs and Fe^3+^ treatments. The gene expression of a Fe^2+^ transporter, Nramp3, was down regulated as well under γ-Fe_2_O_3_ NPs exposure. Although 100 mg/L γ-Fe_2_O_3_ NPs and 20–100 mg/L Fe^3+^ led to higher wax content, genes associated with wax formation (WIN1) and transport (ABCG12) were downregulated or unchanged compared to the control.

**Conclusions:**

Our results showed that both γ-Fe_2_O_3_ NPs and Fe^3+^ exposure via foliar spray had an inconsequential effect on plant growth, but γ-Fe_2_O_3_ NPs can reduce nutrient loss due to their the strong adsorption ability. *C. maxima* plants exposed to γ-Fe_2_O_3_ NPs and Fe^3+^ were in iron-replete status. Moreover, the biosynthesis and transport of wax is a collaborative and multigene controlled process. This study compared the various effects of γ-Fe_2_O_3_ NPs, Fe^3+^ and Fe chelate and exhibited the advantages of NPs as a foliar fertilizer, laying the foundation for the future applications of nutrient-containing nanomaterials in agriculture and horticulture.

**Graphical abstract:**
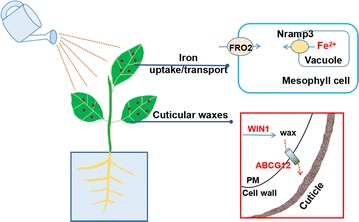
γ-Fe_2_O_3_ NPs exposed on plants via foliar spray and genes associated with the absorption and transformation of iron, as well as wax synthesis and secretion in *Citrus maxima* leaves

**Electronic supplementary material:**

The online version of this article (doi:10.1186/s12951-017-0286-1) contains supplementary material, which is available to authorized users.

## Background

Iron deficiency in plants is widespread and can lead to reduction in crop yields and even complete crop failure [1]. Due to rapid conversion of iron into plant-unavailable forms when applied to calcareous soils, soil application of inorganic iron fertilizers to Fe-deficient soils is usually ineffective [2]. In comparison, synthetic Fe-chelates for amelioration of iron deficiency in plants is more effective, but more uneconomical [3]. It was reported that most foliar-applied micronutrients are not efficiently transported toward roots, which may remain deficient [2]. Nowadays, nanomaterials become a hotspot of research interests and attract the attention of many researchers. A variety of nanoparticles (NPs) have been studied on human cells [4, 5], animal cells [6] and plants [7] about their toxicity or applications. As one of the most widely explored and applied nanomaterials, iron oxide nanoparticles (γ-Fe_2_O_3_ NPs) are widely used in medical diagnostics, controlled drug release, separation technologies and environmental engineering [8]. Iron dynamically released from γ-Fe_2_O_3_ NPs may be a potential nutritional source for plants. It is likely that γ-Fe_2_O_3_ NPs could be an effective fertilizer for alleviation of Fe-deficiency in plants. Several studies have reported that root applied γ-Fe_2_O_3_ NPs have positive effects on plant growth. For instance, γ-Fe_2_O_3_ NPs can physiologically enhance seed germination, root growth, chlorophyll content in watermelon (*Citrullus lanatus*) planted in quartz sand [9] and Chinese mung bean (*Vigna radiata* L.) grown in silica sediment [10]. Rui et al. [11] reported that γ-Fe_2_O_3_ NPs increased root length, plant height, biomass, and chlorophyll levels of peanut (*Arachis hypogaea*) plants, indicating that γ-Fe_2_O_3_ NPs can possibly replace traditional iron fertilizers in the cultivation of peanut plants. To our knowledge, few researchers reported the effects of γ-Fe_2_O_3_ NPs on plants via foliar application yet.

Root is the major pathway for plants to absorb water and inorganic ions [12], through which NPs can be taken up and translocated to upper tissues [13–15]. When NPs were exposed to plants’ leave surface, several studies have observed that plants can absorb NPs through the leaves as well. Corredor et al. [16] reported that carbon coated iron NPs were capable of penetrating pumpkin (*Cucurbita pepo* L.) leaves and migrating to other plant tissues. Larue et al. [17] found that Ag NPs were effectively trapped on lettuce (*Lactuca sativa)* leaves and taken up by cells after foliar exposure. It is hypothesized that there are two pathways for leaves to take up NPs and their solutes: for hydrophilic compounds via aqueous pores of the cuticle and stomata, and for lipophilic ones by diffusion through the cuticle [17]. Since the wax lipids may quickly adsorb on the large surface of NPs [18], particles might be trapped by the cuticular wax and then diffuse in the leaf tissue (after dissolution or translocation through the cuticle) [19]. For example, Birbaum et al. [18] reported that large agglomerates were trapped on the surface wax, whereas smaller particles might be taken up by the leaf. At the molecular level, wax inducer1 (WIN1), an ethylene response factor-type transcription factor, can activate wax deposition in overexpressing plants and influence wax accumulation through the direct or indirect regulation of metabolic pathway genes [20]. Alabdallat et al. [21] reported that WIN1 gene could modulate wax accumulation and enhance drought tolerance in tomato (*Solanum lycopersicum*) plants. Several plant ATP-binding cassette sub-family G member (ABCG) proteins are known or suspected to be involved in synthesis of extracellular barriers, among which ABCG12 is required for lipid export from the epidermis to the protective cuticle [22]. The interactions between γ-Fe_2_O_3_ NPs and plant leaves were inevitably affected by cuticular wax due to the fact that the plant cuticles form the outermost barrier between plant leaves and their local environment. Therefore, it is of great significance to study the changes of cuticular wax in plant leaves induced by foliar sprayed γ-Fe_2_O_3_ NPs. However, to our knowledge, the effects of foliar application of γ-Fe_2_O_3_ NPs on cuticular wax loads and related gene expression have not been reported. In the present study, in order to show the in-depth interactions between γ-Fe_2_O_3_ NPs and cuticular waxes in *Citrus maxima* leaves, wax content and wax synthesis or transport related genes, including WIN1 and ABCG12 were analyzed at the molecular level.

Additionally, in order to figure out the effects of γ-Fe_2_O_3_ NPs on plant growth and physiology, the corresponding parameters, including biomass, chlorophyll, soluble protein content, root activity, lipid peroxidation and activity of antioxidant enzymes were measured. *C. maxima* plants were exposed to 20, 50 and 100 mg/L γ-Fe_2_O_3_ NPs or Fe^3+^ by foliar application at an early growth stage. The latter treatment was set to study the phytotoxicity of Fe^3+^ ions by dissolving FeCl_3_·6H_2_O. This is the first report on the γ-Fe_2_O_3_ NPs uptake and translocation in plants via foliar application, and the transcriptional modulation of genes involved in iron uptake or transport viz. ferric-chelate reductase (FRO2) and natural resistance-associated macrophage protein (Nramp3).

## Methods

### Materials and experimental setups

The γ-Fe_2_O_3_ NPs of 99.5% purity were purchased from Macklin Inc. (Shanghai, China). The shape and size were determined by a Tecnai G2 20 TWIN transmission electron microscope (FEI, USA). The hydrodynamic diameter and zeta potential were determined by a Zetasizer Nano ZS90 dynamic light scattering spectrometer (Malvern Instruments Ltd., United Kingdom). The characteristics of γ-Fe_2_O_3_ NPs are shown in Additional file 1: Figure S1 of the supplementary materials. γ-Fe_2_O_3_ NPs are spherical with an average diameter size of 20.2 ± 2.7 nm (Additional file 1: Figure S1A). The average hydrodynamic diameter and the zeta potential of γ-Fe_2_O_3_ NPs were 164.5 ± 11.3 nm and −11.7 ± 0.1 mV, respectively (Additional file 1: Figure S1B, C). *Citrus maxima* seeds were immersed in distilled water and germinated in moist perlite at 28 °C. Then the uniform seedlings were transferred to a hydroponic system amended with 1/2 Hoagland’s nutrient solution without iron. 18 of seedlings were planted in each hydroponic container. Plants were sprayed with 50 mL of deionized water (control), 20, 50 and 100 mg/L γ-Fe_2_O_3_ NPs suspended in deionized water, 20, 50 and 100 mg/L Fe^3+^ (dissolved from FeCl_3_·6H_2_O) solutions, and 50 μM Fe(II)-EDTA in the morning. During all the treatments, An iron-deficient control and a Fe(II)-EDTA treatment were set up for comparison. The concentrations of Fe^3+^ are calculated according to the containing iron content of γ-Fe_2_O_3_ NPs at same concentration. Therefore, γ-Fe_2_O_3_ NPs and Fe^3+^ treatments marked with the same concentration denote they have same iron content. Suspensions were sprayed with a hand-held sprayer bottle every 5 days when the plants had two true leaves. To facilitate foliar infiltration, all plants were sprayed with deionized water once per hour for 10 h to avoid early evaporation of the solutions and consequent precipitation of solutes on the leaf surface [23]. The plants were grown in an environmentally controlled growth chamber at 28/18 °C with a 16 h/8 h light/dark cycle; the light intensity was 2000 lx. The air was pumped into the hydroponic system every 3 h with 30 min each time. The nutrient solution was replaced every 5 days. After 30 days of exposure, representative parameters including chlorophyll, fresh biomass, soluble protein content, root activity, lipid peroxidation, antioxidant enzyme activities, iron content, iron-related gene expression, wax content, and wax-related gene expression were measured.

### Fresh biomass measurement

*Citrus maxima* plants were carefully removed from the hydroponic system after 30 days. The fresh biomass of *C. maxima* including roots and shoots was weighed by using a FA1004C electronic analytical balance (Shanghai Yueping Scientific Instrument Co., Ltd, China).

### Measurement of physiological and biochemical parameters

Chlorophyll content was determined by a modified procedure according to Lichtenthaler [24]. Soluble protein content was estimated according to a dying method using Coomasie Brilliant Fluka G-250 [10]. Measurement of root activity was according to the triphenyltetrazolium chloride method [25]. Malonaldehyde (MDA) was determined by the thiobarbituric acid method according to Heath and Packer [26]. The activity of superoxide dismutase (SOD) was evaluated by the ability to inhibit photochemical reduction of nitroblue tetrazolium according to Wang et al. [27]. The activity of catalase (CAT) was analyzed as described by Gallego et al. [28]. The activity of peroxidase (POD) was estimated by guaiacol colorimetric method as described by Zhang et al. [29].

### Metal uptake analysis

Harvested leaf tissue was rinsed with deionized H_2_O thrice to remove the surface retained γ-Fe_2_O_3_ NPs. All shoot and root samples were dried at 60 °C for 48 h in a drying oven. 100 mg of oven-dried shoot and root tissues were separately digested in 3 mL of concentrated HNO_3_ at 115 °C on a hot block for 1 h. After cooling to room temperature, 0.5 mL of 30% H_2_O_2_ was added to the digestions at 100 °C for 0.5 h. The iron content was analyzed by an Avanta M atomic absorption spectrophotometer (GBC, Australia).

### Measurement of wax loads

The content of cuticular waxes was determined using chloroform extraction as described by Premachandra et al. [30]. Leaf samples were immersed in 20 mL of chloroform in a Petri dish of 90 mm diameter for 5 s. The solvent was evaporated in a fume hood under a dry air stream, and the residue was allowed to dry for 24 h at room temperature. After drying, the content of cuticular waxes was weighed by using a FA1004C electronic analytical balance and expressed on the basis of FW (fresh weight).

### Regulation of gene expression by RT-PCR

The isolation of total RNA, the synthesis of cDNA and RT-PCR analysis were conducted according to our previous study [31]. Primers for FRO2, Nramp3, ABCG12 and WIN1 genes were designed based on the sequences available in NCBI genbank using the PrimerQuest (Integrated DNA Technologies, Coralville, IA) as described in Table 1.

**Table 1:** Primers of genes used in this study

Gene	Primer sequence (Forward-5′–3′)	Primer sequence (Reverse-5′–3′)
Actin	CAGCTGTGGAGAAGAGCTATG	CGATCATGGATGGTTGGAAGA
Nramp3	GCGTGTTGATTGCTACTGTTATT	GATGAGCACGCCAACTAGAA
FRO2	GTGTCTGTTGAAGGACCCTATG	GCTCGCGGACTATGGAAATAA
ABCG12	GGAAGGGCTGGAAATTGAAATC	GCCCAGTAATATCCCACATCTC
WIN1	GCTCCTCATCATCATCACCTAC	GCCTCAGACAAGTCATAGAAGG

### Statistical analysis

Each treatment was conducted with three replicates, and the results were presented as mean ± SD (standard deviation). The statistical analysis of experimental data was verified with the one-way ANOVA followed by Duncan’s multiple comparison (*p* < 0.05) in the statistical package IBM SPSS Version 22.

## Results

### Effect of γ-Fe_2_O_3_ NPs and Fe^3+^ treatments on plant growth

**Fig. 1:**
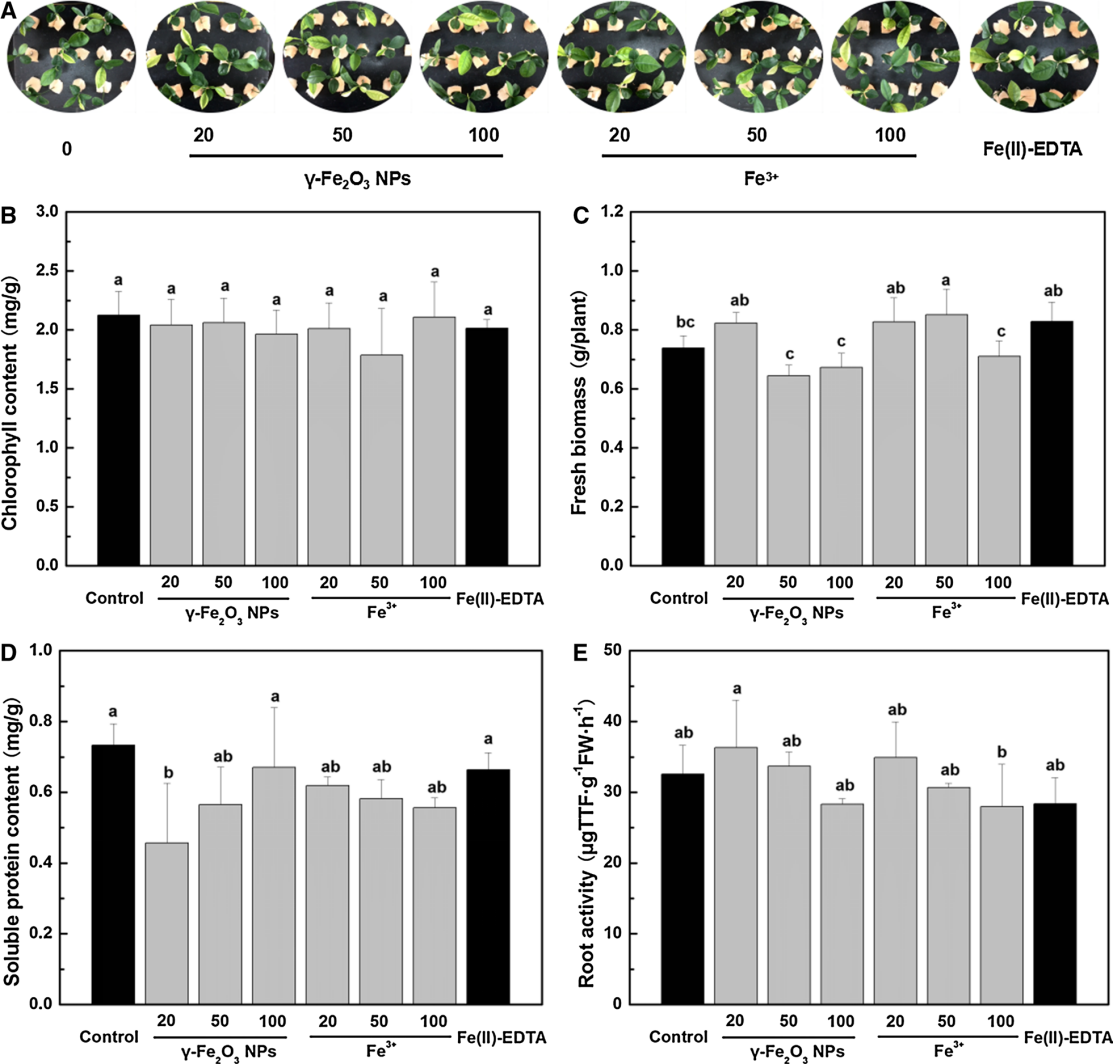
**A** Images of *C. maxima* leaves exposed to different concentrations of γ-Fe_2_O_3_ NPs and Fe^3+^. **B**–**E** Chlorophyll content, fresh biomass, soluble protein content in leaves, and root activity of *C. maxima* plants treated with different concentrations of γ-Fe_2_O_3_ NPs and Fe^3+^, respectively. Data are shown as mean ± SD of three replicates. Values followed by *different lowercase letters* are significantly different at *p* ≤ 0.05

The influence of γ-Fe_2_O_3_ NPs and their counterpart Fe^3+^ solutions (20–100 mg/L) on the growth of *C. maxima* leaves is shown in Fig. 1A. No visible signs of phytotoxicity are evident in *C. maxima* leaves under all treatments. As show in Fig. 1B, chlorophyll contents of all treatments showed no significant differences. The fresh biomass of Fe-exposed *C. maxima* plants had no significant differences from that of the control, except for 50 mg/L Fe^3+^ treatment, which had 15.4% higher fresh biomass (Fig. 1C). On the other hand, no positive effect of fresh biomass under the exposure of γ-Fe_2_O_3_ NPs and Fe^3+^ was induced compared with Fe(II)-EDTA treatment. Instead, fresh biomass of *C. maxima* seedlings was decreased by 22.1, 18.7 and 14.3% under the exposure of 50 and 100 mg/L γ-Fe_2_O_3_ NPs, and 100 mg/L Fe^3+^, respectively.

Soluble protein amounts at various concentrations of γ-Fe_2_O_3_ NPs and Fe^3+^ exposure were unaffected compared to the control and Fe(II)-EDTA treatment, except for 20 mg/L γ-Fe_2_O_3_ NPs, which had lower soluble protein content (Fig. 1D). Root activity is a comprehensive assessment index that reflects the metabolic activity level and the ability of roots to absorb nutrients and water [32]. As Fig. 1E depicted, all foliar applied γ-Fe_2_O_3_ NPs and Fe^3+^ treatments had no impact on root activity as compared to the control and Fe(II)-EDTA treatment.

### Lipid peroxidation and antioxidant enzyme activities of *C. maxima* plants

**Fig. 2:**
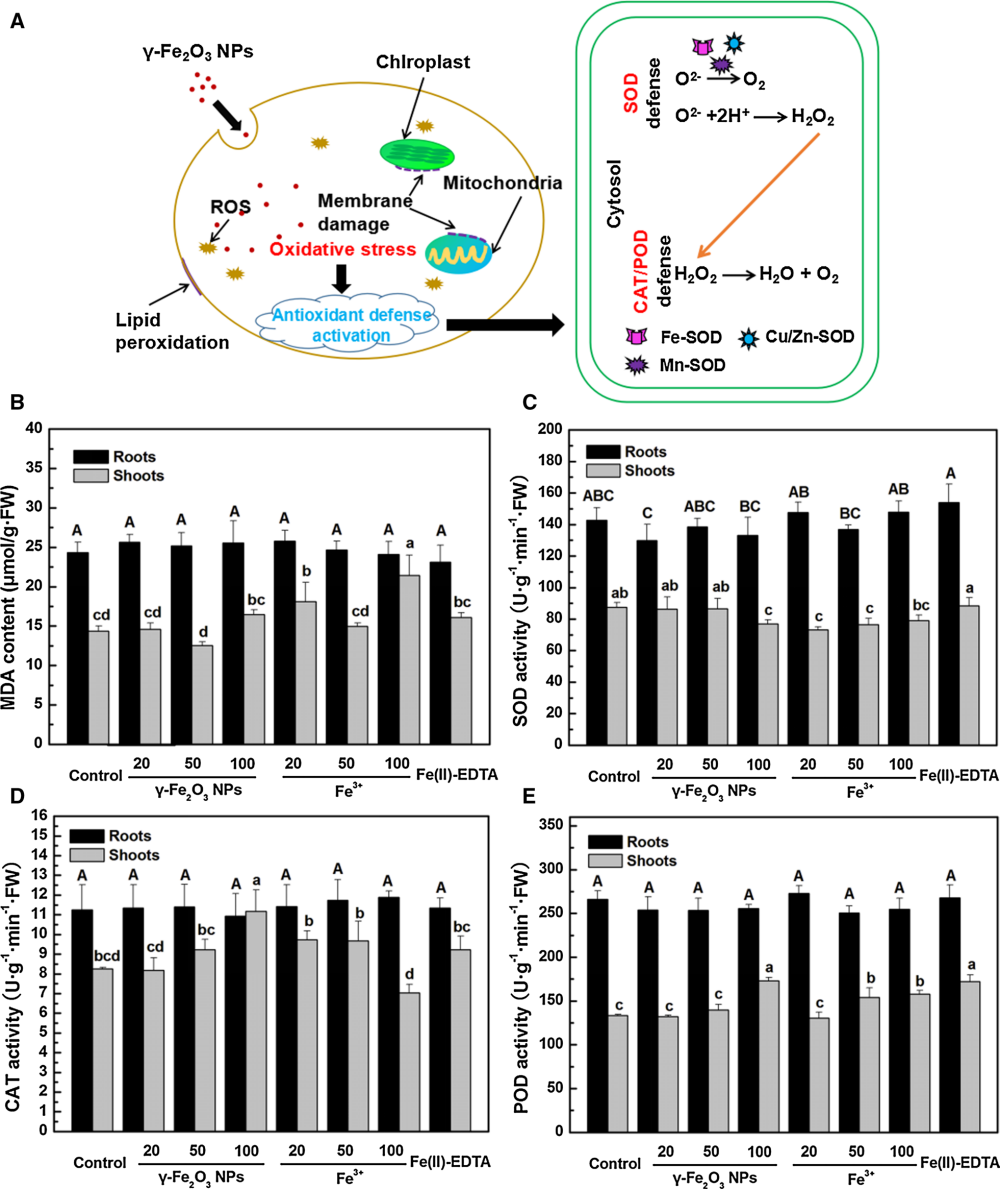
**A** Schematic illustration of the activation of antioxidant enzymes in plants to scavenge excessive ROS production induced by γ-Fe_2_O_3_ NPs. **B**–**E** MDA content, activity of SOD, CAT and POD in roots and shoots of *C. maxima* plants treated with different concentrations of γ-Fe_2_O_3_ NPs and Fe^3+^, respectively. Data are shown as mean ± SD of three replicates. Values of MDA content and antioxidant enzyme activities followed by *different lowercase* and *uppercase letters*, respectively, are significantly different at *p* ≤ 0.05

The oxidative stress induced by γ-Fe_2_O_3_ NPs and subsequent reactive oxygen species (ROS) scavenging by SOD, CAT and POD are presented schematically in Fig. 2A. In *C. maxima* shoots, no elevated lipid peroxidation by γ-Fe_2_O_3_ NPs was observed compared to both the control and Fe(II)-EDTA treatment (Fig. 2B). 20 and 100 mg/L Fe^3+^ treatment had a higher MDA formation by 26.0 and 49.1%, respectively as compared with the control. Also, MDA level of 100 mg/L Fe^3+^ treatment was 33.2% higher than Fe(II)-EDTA treatment. In *C. maxima* roots, MDA production remained unchanged regardless of the treatments.

Compared with the control and Fe(II)-EDTA treatment, the activities of SOD did not increase in both *C. maxima* shoots and roots after the γ-Fe_2_O_3_ NPs and Fe^3+^ treatments (Fig. 2C). As depicted in Fig. 2D, CAT activity of γ-Fe_2_O_3_ NPs treated *C. maxima* shoots numerically increased in a dose-dependent manner. Statistically, 100 mg/L γ-Fe_2_O_3_ NPs had 35.4% higher CAT activity than the control, and 21.1% higher than Fe(II)-EDTA treatment. On the other hand, CAT activity in Fe^3+^-treated shoots was not significantly different from the control, but 100 mg/L Fe^3+^ treatment resulted in 31.0% lower CAT activity than Fe(II)-EDTA treatment. POD activities in shoots treated with 20 and 50 mg/L γ-Fe_2_O_3_ NPs, and 20 mg/L Fe^3+^ treatment were unaffected, while those of 100 mg/L γ-Fe_2_O_3_ NPs, 50 and 100 mg/L Fe^3+^ treatment were increased significantly, as compared to the control (Fig. 2E). In addition, no increase of POD activity in shoots under γ-Fe_2_O_3_ NPs or Fe^3+^ treatments was observed compared to that of Fe(II)-EDTA treatment. In *C. maxima* roots, both the CAT and POD activities remained unchanged, no matter what concentrations of γ-Fe_2_O_3_ NPs or Fe^3+^were used (Fig. 2D, E).

### Iron distribution and iron-related gene expression in *C. maxima* plants

**Fig. 3:**
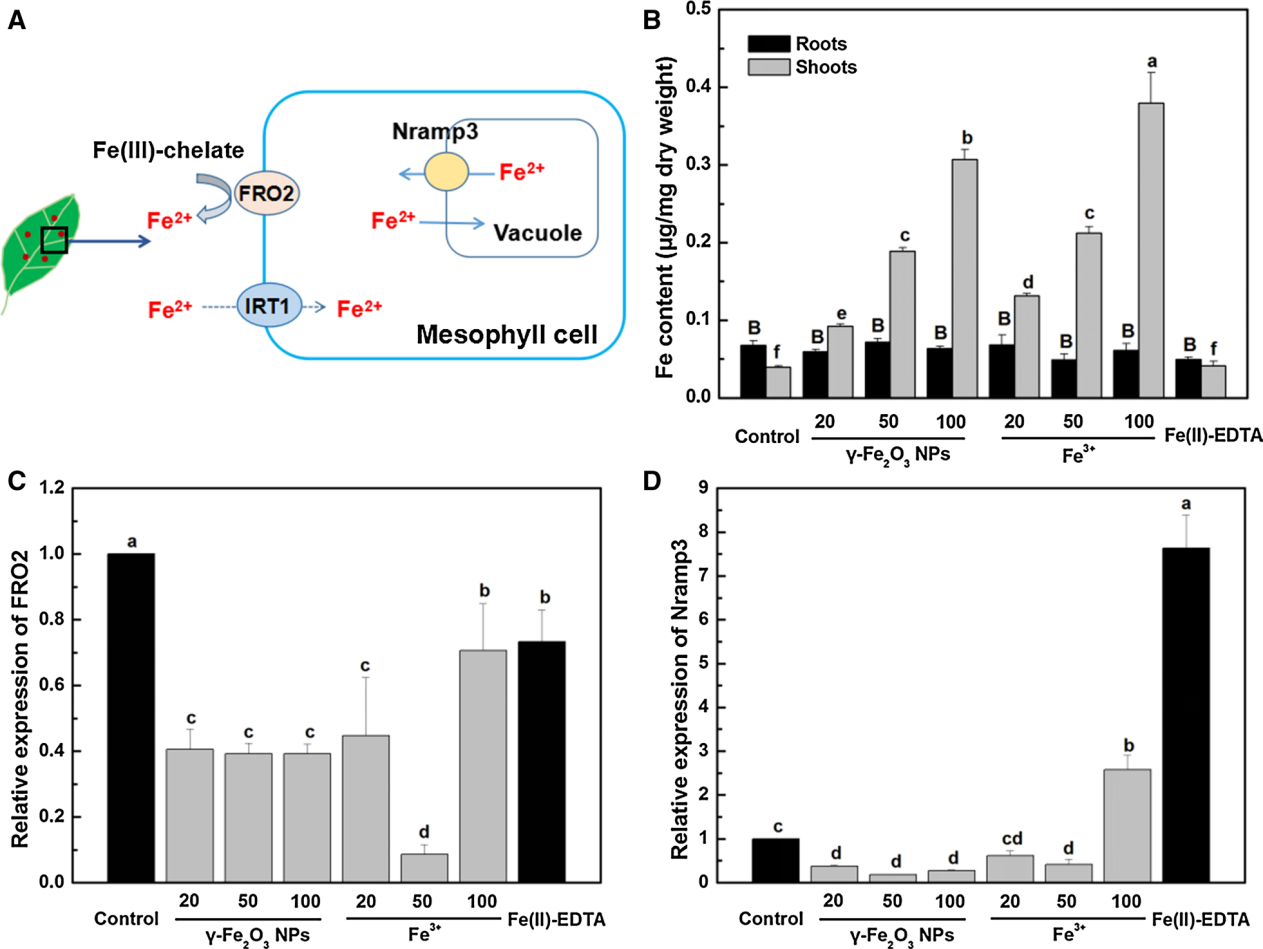
**A** Schematic diagram of genes associated with the absorption and transformation of iron in plant leaves. **B**–**D** Iron content of *C. maxima* including roots and shoots, relative expression of FRO2 and Nramp3 of *C. maxima* leaves treated with different concentrations of γ-Fe_2_O_3_ NPs and Fe^3+^, respectively. Data are shown as mean ± SD of three replicates. Values of Fe content and relative expression of each gene labelled by *different lowercase* and *uppercase letters*, respectively, are significantly different at *p* ≤ 0.05

The possible pattern of transformation and uptake of iron in *C. maxima* leaves is shown in Fig. 3A. Unfortunately, iron regulated transporter (IRT1) gene in citrus has not been sequenced yet. As expected, Fe concentration of *C. maxima* shoots exposed to both γ-Fe_2_O_3_ NPs and Fe^3+^ treatment increased rapidly with the increase of applied dosages (Fig. 3B). After exposure to 20, 50 and 100 mg/L γ-Fe_2_O_3_ NPs, Fe content in shoots was increased by 1.34, 3.78 and 6.77 times, respectively, relative to the control plants. Fe level of Fe^3+^ treatments was elevated by 2.33, 4.38, 8.62 times, respectively. In addition, the total Fe content in *C. maxima* shoots was not significantly different between Fe(II)-EDTA and control plants. In *C. maxima* roots, no obvious difference of Fe levels was noted between all Fe treatments and control plants.

FRO2 gene encodes a ferric chelate reductase, which can be activated when plants lack available Fe. As seen in Fig. 3C, the relative FRO2 gene expression of control was at a high level. γ-Fe_2_O_3_ NPs and Fe^3+^ treatments led to 29.4–91.4% lower levels of FRO2 gene expression than that of untreated control plants. Especially, 50 mg/L Fe^3+^ treatment significantly decreased FRO2 expression to a much lower level than other treatments. Meanwhile, FRO2 expression level of Fe(II)-EDTA treatment was also lower than untreated control, but not less than that of γ-Fe_2_O_3_ NPs and Fe^3+^ treatments. Nramp3 protein, which localizes in the vacuolar membrane (Fig. 3A), can transport Fe^2+^ and is upregulated by iron starvation. As depicted in Fig. 3D, 20–100 mg/L γ-Fe_2_O_3_ NPs had relatively lower expression levels of Nramp3 gene than control by 62.5–81.7%, but that of 100 mg/L of Fe^3+^ treatment was much higher by 1.58 times. Interestingly, Fe(II)-EDTA treatment had a quite higher level of Nramp3 gene expression.

### Wax content and wax-related gene expression of *C. maxima* leaves

**Fig. 4:**
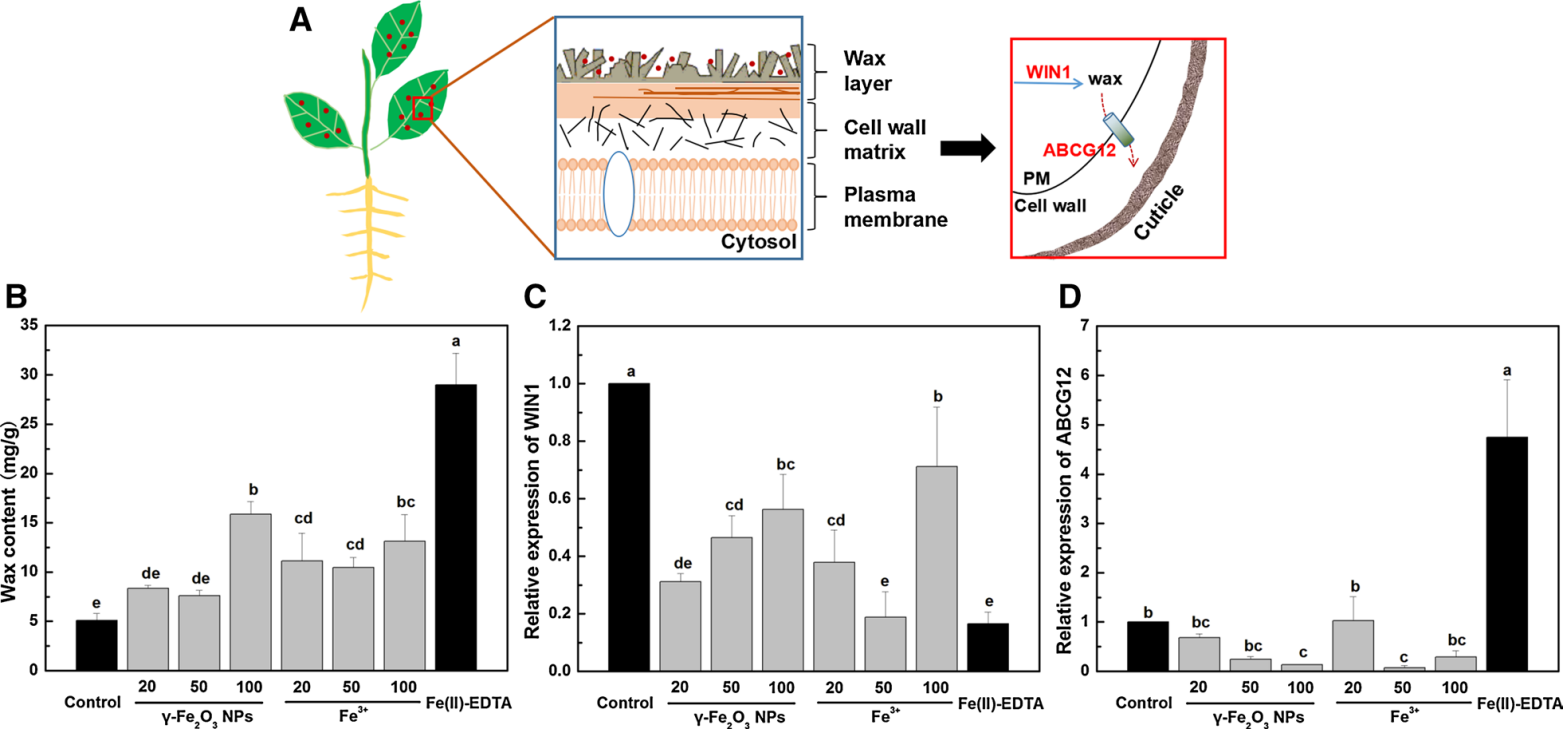
**A** Schematic diagram of the interactions between NPs and cuticular waxes in leaves, and genes involved in wax synthesis and secretion in this study (PM: plasma membrane). **B**–**D** represent wax content, relative expression of WIN1 and ABCG12 genes of *C. maxima* leaves treated with different concentrations of γ-Fe_2_O_3_ NPs and Fe^3+^, respectively. Data are shown as mean ± SD of three replicates. Values of wax content and relative expression of each gene followed by *different lowercase letters* are significantly different at *p* ≤ 0.05

The potential interactions between γ-Fe_2_O_3_ NPs and cuticular wax as well as genes involved in the intracellular wax synthesis and transport to the outside of cell walls are presented schematically in Fig. 4A. Wax, which is composed of long-chain, aliphatic hydrocarbons derived from very-long-chain fatty acids (VLCFAs) [33], is the protective material on leaf epidermis [34] and plays an important role in particle incorporation. As seen from Fig. 4B, 20 and 50 mg/L γ-Fe_2_O_3_ NPs had no impact on wax content compared with the control, while 100 mg/L γ-Fe_2_O_3_ NPs exhibited significantly higher wax content by 2.1-fold. 20, 50 and 100 mg/L Fe^3+^ treatment had higher wax contents than the control by 1.17, 1.04 and 1.57 times, respectively. Wax content of Fe(II)-EDTA treatment was in a notably higher level compared with other treatments.

The relative expression levels of WIN1 gene under the exposure of all γ-Fe_2_O_3_ NPs and Fe^3+^ treatments were significantly lower than the control but not less than that of Fe(II)-EDTA treatment (Fig. 4C). In Fig. 4D, the relative expression levels of ABCG12 gene treated by γ-Fe_2_O_3_ NPs and Fe^3+^ were lower or unaffected as compared to untreated control. However, Fe(II)-EDTA treatment had a much higher ABCG12 gene expression level by contrast with other treatments.

## Discussion

### Growth and physiological effects of γ-Fe_2_O_3_ NPs and Fe^3+^

Fe(II)-EDTA, as one of the most widely used supplements for improving Fe availability to plants [35], showed no evident promotion to plant growth via foliar application in our study. Meantime, γ-Fe_2_O_3_ NPs did not exhibit any superiority in overcoming Fe deficiency-induced chlorosis. We did not observe any evident difference of chlorophyll levels among treatments of γ-Fe_2_O_3_ NPs, Fe^3+^, control and Fe(II)-EDTA, although iron content in *C. maxima* shoots of γ-Fe_2_O_3_ NPs and Fe^3+^ treatments was higher than control and Fe(II)-EDTA treatment. It is possible that iron was mainly used in other physiological reactions and thus no significant changes in chlorophyll content were observed. It is noteworthy that previously we found that through root exposure in a hydroponic system, 0–100 mg/L of γ-Fe_2_O_3_ NPs and Fe^3+^ had a dose-dependent effect on chlorophyll synthesis of *C. maxima* [31]. 50 mg/L γ-Fe_2_O_3_ NPs and all Fe^3+^ treatments notably increased chlorophyll levels. Fe(II)-EDTA treatment also had higher chlorophyll content as compared to the untreated control. However, foliar applications of γ-Fe_2_O_3_ NPs and Fe^3+^, as well as Fe(II)-EDTA, appeared to have no positive effect on chlorophyll synthesis and no obvious amelioration of chlorosis was observed, indicating that foliar application was less efficient than root application. However, Alidoust and Isoda [23] observed more pronounced positive effects of γ-Fe_2_O_3_ NPs on physiological performance of soybean (*Glycine max* (L.) Merr.) via foliar application than by soil treatment. They used different parameters, including γ-Fe_2_O_3_ NP size and concentrations, growth condition, treatment time as well as plant species, which may explain the contradictory results from ours.

To demonstrate if γ-Fe_2_O_3_ NPs altered the plant health at physiological level, we analyzed the change of soluble proteins, which is an important indicator of plants’ defense. Plants could adapt themselves to various stresses by producing soluble proteins as osmolytes [36], antioxidants, or scavengers for eliminating free radicals in plants [37]. For example, Afaq et al. [38] observed an increase in the antioxidant enzymes after TiO_2_ NPs treatment as indicated at the transcriptional or protein level. Meanwhile, it is known that various abiotic stresses lead to the overproduction of ROS in plants which are highly reactive and toxic, ultimately resulting in oxidative stress and protein damage [39]. Nevertheless, in this study, no oxidative stress was induced in plants exposed to 20 mg/L γ-Fe_2_O_3_ NPs, based on the results of MDA content and the antioxidant enzyme activities (Fig. 2B–E), indicating that the lower soluble protein level could be caused by an alternative mechanism, instead of protein damage caused by overproduction of ROS. Meantime, the unchanged soluble protein contents under other treatments might be a result of self-regulation by plants.

### Oxidative stress caused by γ-Fe_2_O_3_ NPs and Fe^3+^ on plants

In this study, no elevated MDA level in shoots under γ-Fe_2_O_3_ NPs exposure was induced, suggesting that either foliar applied γ-Fe_2_O_3_ NPs did not induce lipid peroxidation even at high exposure concentrations or the plant’s detoxification pathways were sufficient to address and remedy the induced stress [40]. Activities of three antioxidant enzymes in plants treated with 20 and 50 mg/L γ-Fe_2_O_3_ NPs were unaffected, while 100 mg/L γ-Fe_2_O_3_ NPs significantly increased the activity of CAT and POD. Higher activity of CAT and POD can contribute to the detoxification of excessive amounts of H_2_O_2_ [41]. Given the results of MDA levels and antioxidant enzymes, it is clear that 20 and 50 mg/L γ-Fe_2_O_3_ NPs did not induce oxidative stress in plant shoots, while 100 mg/L γ-Fe_2_O_3_ NPs might initially cause ROS generation but then the plant’s defense systems remedied the induced stress. As for Fe^3+^ treatments, 20 and 100 mg/L treated shoots showed a much higher MDA content, while that of 50 mg/L Fe^3+^ treatment was unaffected compared with the control. However, no elevated activities of three antioxidant enzymes under 20 mg/L Fe^3+^ treatment were observed, indicating that the increase of MDA level under 20 mg/L Fe^3+^ was an abnormal result. Combined MDA content with the higher POD activity of 50 and 100 mg/L Fe^3+^ treatments in shoots, it could be deduced that *C. maxima* treated with 50 mg/L Fe^3+^ could address and remedy the induced oxidative stress, while plants treated with 100 mg/L Fe^3+^ were not sufficient to deal with stress induced by Fe^3+^ at a high concentration. The unchanged MDA production and antioxidant enzyme activities in *C. maxima* roots among all the treatments indicated that no oxidative stress occurred in plant roots.

### Uptake and translocation of γ-Fe_2_O_3_ NPs

Iron content of *C. maxima* shoots exposed to different concentrations of γ-Fe_2_O_3_ NPs showed a dose-dependent trend. The higher Fe level of γ-Fe_2_O_3_ NPs in shoots indicated that significant uptake had occurred. Several studies demonstrated that Fe_2_O_3_ NPs in a hydroponic system could enter plants through roots [42, 43], or silica sediment [10]. However, to our knowledge, few studies investigated whether foliar applied γ-Fe_2_O_3_ NPs could enter plant leaves and further translocate to roots or not. We observed the uptake of iron into shoots but no difference of iron content in *C. maxima* roots between all treatments, suggesting that no downward transport of iron occurred in *C. maxima* plants. In our previous study, we observed that root-applied γ-Fe_2_O_3_ NPs had no translocation from roots to shoots [31]. Therefore, either foliar spray or root supply of γ-Fe_2_O_3_ NPs alone cannot meet the requirement of the whole plants. A combination of both application methods may improve the effectiveness of iron fertilization in agricultural and horticultural production.

Generally, when plants are deprived of iron, the new leaves become chlorotic and young lateral roots show the characteristic Fe-deficient stress-response mechanisms: enhanced Fe(III) reducing capacity, subapical swelling and acidification of the medium [44]. However, previous studies showed that leaf mesophyll cells also display plasma membrane ferric reductase activity [44, 45]. When a plant suffers from iron shortage, the reductive system is strongly activated [45], with FRO2 gene encoding a ferric chelate reductase. The down-regulation of FRO2 gene expression under γ-Fe_2_O_3_ NPs and Fe^3+^ treatments compared to the control and Fe(II)-EDTA treatment indicated that *C. maxima* could utilize the supplied iron in γ-Fe_2_O_3_ NPs and Fe^3+^ via foliar application. The relative level of FRO2 expression exposed to 50 mg/L Fe^3+^ was the lowest. The supply of iron is not only dependent on applied dosage, but also plants’ utilization ability. Based on the MDA data, 20 and 100 mg/L Fe^3+^ treatments had higher MDA formations than 50 mg/L Fe^3+^, which indicates that 50 mg/L leads to less oxidative stress than the other two dosages. Therefore, plants could better utilize Fe^3+^ at 50 mg/L, which explains why the activation of FRO2 gene of 50 mg/L was lower than 20 and 100 mg/L Fe^3+^ treatments. Taken together, 50 mg/L Fe^3+^ can supply higher amount of iron than 20 mg/L Fe^3+^. Meanwhile, the toxicities of 20 mg/L and 100 mg/L Fe^3+^ are higher than that of 50 mg/L and likely inhibits the leaf ability to absorb and utilize iron. Also, γ-Fe_2_O_3_ NPs and Fe^3+^ had a higher ability to supply iron to plants than Fe(II)-EDTA, except for 100 mg/L Fe^3+^. In addition, the lower level of Nramp3 gene expression at all γ-Fe_2_O_3_ NPs concentrations indicated that plant was in iron-sufficient status. The much higher level of Nramp3 gene expression of 100 mg/L Fe^3+^ and Fe(II)-EDTA treatment than the control suggested that Fe(II)-EDTA and Fe^3+^ at high concentrations cannot alleviate iron deficiency via foliar spray. It was reported that Fe-chelates are more effective in soil than in foliar applications, and foliar Fe chelate-fertilization cannot yet be considered as a reliable strategy to control plant Fe-deficiency [46]. Previous study showed that γ-Fe_2_O_3_ NPs is a suitable adsorbent for effectively extracting pollutants from the environment due to their high specific surface area and accessible surface adsorption sites, which make them well applicable for the adsorption of pollutants [47, 48]. Given this, the strong adsorption ability of γ-Fe_2_O_3_ NPs contributed to their stable attachment on the leaf surface and further absorption by plants. In agricultural production, most of the applied fertilizers are frequently lost due to the degradation by photolysis, leaching, hydrolysis, and decomposition [49]. It is essential to reduce nutrient losses in fertilization and increase the crop yield through the development of nanomaterials-based fertilizers [49]. In this regard, our results revealed that γ-Fe_2_O_3_ NPs have the potential to be an effective nanofertilizer and reduce nutrient loss during and after application.

### Interaction between γ-Fe_2_O_3_ NPs and cuticular wax

In this study, iron distribution indicated that γ-Fe_2_O_3_ NPs may be tightly attached to the leaf surface and/or taken up by the *C. maxima* leaves. Cuticular wax is a protective barrier on leaf epidermis, which could adsorb and trap intrusive NPs. Once NPs translocate through the cuticle, NPs could diffuse in the leaf tissue. We observed a significantly lower expression levels of WIN1 gene under all Fe exposures. No upregulation of ABCG12 gene expression treated with γ-Fe_2_O_3_ NPs and Fe^3+^ treatments was observed as well. However, wax contents of 100 mg/L γ-Fe_2_O_3_ NPs and 20–100 mg/L Fe^3+^ treatment were significantly enhanced. Such an increase of wax content could hinder the uptake of high levels of γ-Fe_2_O_3_ NPs and ionized iron (Fe^3+^). Wax content is closely correlated with stress resistance of plants [50]. According to Fig. 2B–E, Fe^3+^ treatments induced stress in plant shoots. The higher wax levels of plant leaves under Fe^3+^ treatments might be a result of anti-stress. The high Fe content in shoots of 100 mg/L γ-Fe_2_O_3_ NPs suggested that most NPs were trapped on the surface wax as a result of the formation of clusters and large agglomerates [18]. Strangely, Fe(II)-EDTA treatment had a lower WIN1 gene expression level but a much higher ABCG12 gene expression level, while wax content of Fe(II)-EDTA treatment was at a notably high level. Fernández et al. [46] reported that sprayed Fe-chelates could be taken up via the cuticle due to the comparable sizes of Fe-compounds and the pores. The significantly higher wax content of Fe(II)-EDTA might be a mechanism of defending against alien substances. In addition to WIN1, there are many genes involved in the synthesis and secretion of surface wax [51]. For instance, CUT1, an Arabidopsis gene required for cuticular wax production, encodes a VLCFA condensing enzyme [33]. Therefore, the biosynthesis of wax is a collaborative and complicated process, which explain why 100 mg/L γ-Fe_2_O_3_ NPs and 20–100 mg/L Fe^3+^ led to higher wax content without inducing higher expression levels of WIN1 and ABCG12, as well as Fe(II)-EDTA treatment had the lower expression of WIN1 but higher level of wax. Moreover, Jetter et al. [52] reported that cuticular wax is typically a complex mixture of dozens of compounds with diverse hydrocarbon chain or ring structures. How much each of the wax compounds contributes to the overall biological functions of the cuticular wax is largely unknown [53]. Therefore, further explorations should be made to figure out the processes and mechanisms underlying the interactions between NPs and cuticular waxes.

## Conclusions

Based on the growth and physiological parameters, it is clear that foliar sprayed γ-Fe_2_O_3_ NPs and Fe^3+^ at the concentrations used in this study had an inconsequential effect on plant growth as shown in chlorophyll content, fresh weight, and root activity. However, the expression of genes associated with the absorption and transformation of iron in leaves showed that plants were in iron-sufficient status. Further analysis of iron content shows no downward transport of iron from shoots to roots in all treated forms via foliar application. It is well known that iron is hard to transport from leaves. As for lipid peroxidation, all γ-Fe_2_O_3_ NPs exposures showed insignificant changes as compared with the control. Antioxidant analysis indicated that 20 and 50 mg/L γ-Fe_2_O_3_ NPs induced no oxidative stress while 100 mg/L γ-Fe_2_O_3_ NPs may induced stress initially but plants were sufficient to deal with it. Moreover, the higher wax content of 100 mg/L γ-Fe_2_O_3_ NPs as compared with the control would hinder the uptake of high levels of γ-Fe_2_O_3_ NPs. Results of WIN1 and ABCG12 gene expression revealed that the biosynthesis of wax is a collaborative and complicated process and more than one gene are involved in this process. Commendably, foliar applied γ-Fe_2_O_3_ NPs have the ability to reduce nutrient loss probably due to the strong adsorption ability and gradual Fe release. Given that no phytotoxicity of γ-Fe_2_O_3_ NPs at lower concentrations (20 and 50 mg/L) was observed, it is possible that using γ-Fe_2_O_3_ NPs at lower doses is feasible to enhance the utilization and efficiency of inorganic iron fertilizer in agricultural production. Moreover, in real applications, foliar sprayed γ-Fe_2_O_3_ NPs may be utilized together with soil supplied γ-Fe_2_O_3_ NPs to alleviate chlorosis and improve the iron use efficiency. Our findings provide a novel perspective to the interactions between foliar-applied NPs and plants, and will inspire further critical efforts to systemically explore the potential applications of γ-Fe_2_O_3_ NPs in agronomic production.

There is still much unknown about the speciation change of γ-Fe_2_O_3_ NPs during plant foliar interactions. Further efforts should be made to determine (1) if the γ-Fe_2_O_3_ NPs are absorbed as NPs directly, or dissolution occurs inside or outside plant leaves with free iron ions available for uses by plant leaves; (2) if γ-Fe_2_O_3_ NPs pass through leaf epidermis as NPs, what is their final speciation after interacting with leaf organelles? In addition, 20 mg/L may not be the lowest concentration to supply sufficient iron for plants. Concentrations of γ-Fe_2_O_3_ NPs lower than 20 mg/L should be tested in the future.

## Additional file

**Additional file 1: Figure S1.** (A) TEM image showing the morphology of the suspension of γ-Fe_2_O_3_ NPs in deionized water. DLS analysis showing (B) size distribution and (C) zeta potential of γ-Fe_2_O_3_ NPs in deionized water.

## Abbreviations

ABCG: ATP-binding cassette sub-family G member
CAT: catalase
FRO: ferric-chelate reductase
FW: fresh weight
IRT: iron regulated transporter
MDA: malonaldehyde
NPs: nanoparticles
Nramp: natural resistance-associated macrophage protein
PM: plasma membrane
POD: peroxidase
ROS: reactive oxygen species
SOD: superoxide dismutase
WIN: wax inducer
