# Supplementary material for: Interaction of γ-Fe2O3 nanoparticles with Citrus maxima leaves and the corresponding physiological effects via foliar application
Source: J Nanobiotechnology. 2017 Jul 11;15:51. doi: 10.1186/s12951-017-0286-1 (PMC5504858; doi:10.1186/s12951-017-0286-1)
Supplement: Supplementary file 1 — Additional file 1: Figure S1. (A) TEM image showing the morphology of the suspension of γ-Fe2O3 NPs in deionized water. DLS analysis showing (B) size distribution and (C) zeta potential of γ-Fe2O3 NPs in deionized water. [file 12951_2017_286_MOESM1_ESM.doc]

**Additional file**

**
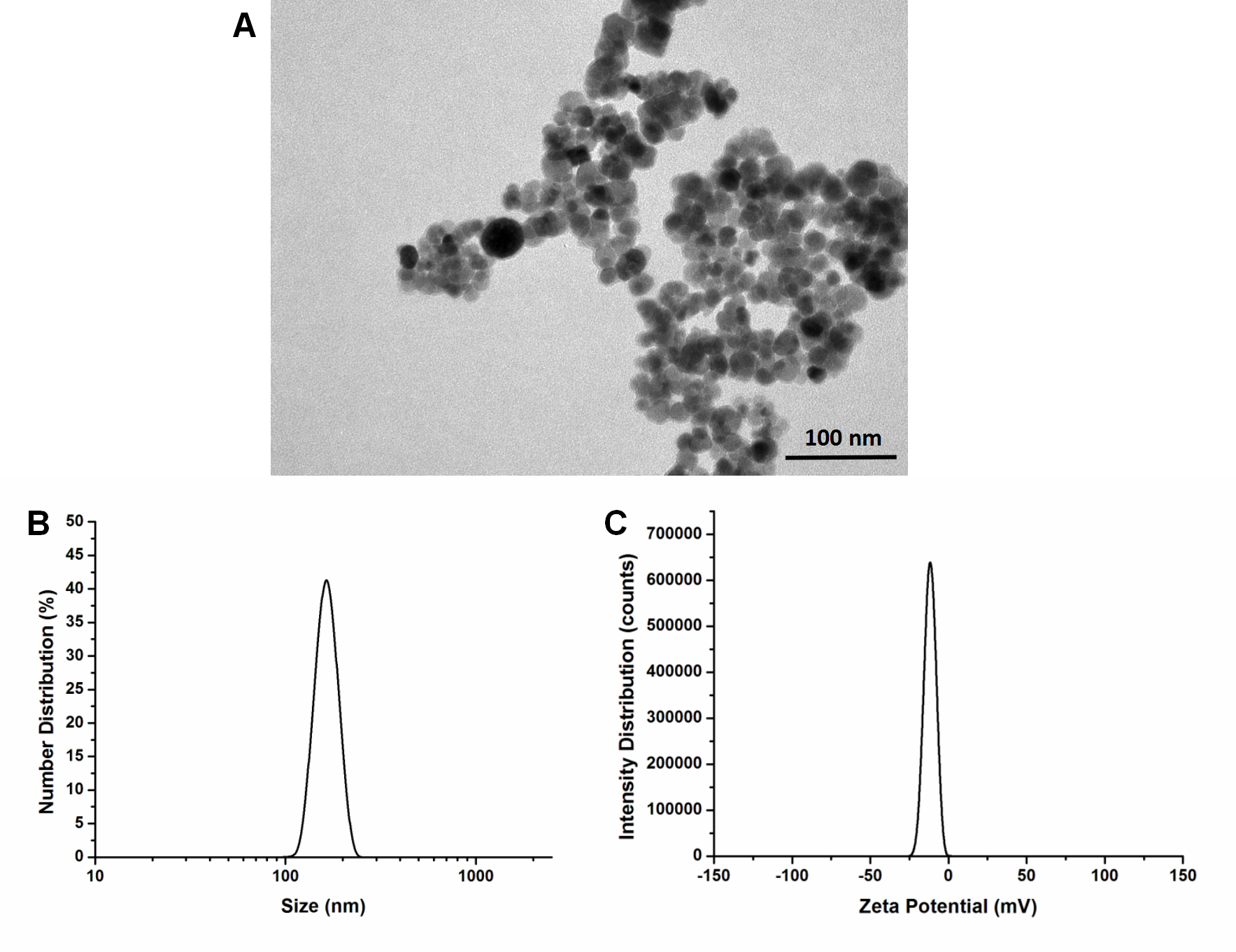
**

**Fig. S1** (A) TEM image showing the morphology of the suspension of γ-Fe_2_O_3_ NPs in deionized water. DLS analysis showing (B) size distribution and (C) zeta potential of γ-Fe_2_O_3_ NPs in deionized water.
